# Low‐Grade Papillary Urothelial Carcinoma of the Bladder Presenting as Persistent Genital Arousal Disorder/Genito‐Pelvic Dysesthesia: A Case Report

**DOI:** 10.1155/criu/3816720

**Published:** 2026-05-25

**Authors:** Sarah Ponce, Eliza Burr, Melissa Davide, Évéline Poirier, Marta Kolbuszewska, Shari McKinny, Rachel S. Rubin, Barry R. Komisaruk, Irwin Goldstein

**Affiliations:** ^1^ Department of Medical Education, Keck School of Medicine of USC, Los Angeles, California, USA, usc.edu; ^2^ Department of Internal Medicine, Beth Israel Deaconess Medical Center, Boston, Massachusetts, USA, bidmc.org; ^3^ Department of Medical Education, Hackensack Meridian School of Medicine, Nutley, New Jersey, USA, hmsom.org; ^4^ Department of Psychology, Queens University, Kingston, Ontario, Canada, queensu.ca; ^5^ Department of Psychology, University of British Columbia, Vancouver, British Columbia, Canada, ubc.ca; ^6^ Department of Urology, University of Saskatchewan, Saskatoon, Saskatchewan, Canada, usask.ca; ^7^ Department of Urology, Medstar Georgetown University Hospital, Washington, DC, USA, medstargeorgetown.org; ^8^ Department of Rutgers University-Newark, Newark, New Jersey, USA; ^9^ San Diego Sexual Medicine, San Diego, California, USA

**Keywords:** bladder cancer, case report, genito-pelvic dysesthesia, persistent genital arousal disorder

## Abstract

Persistent genital arousal disorder/genito‐pelvic dysesthesia (PGAD/GPD) is a rare and distressing condition characterized by unwanted genital arousal without associated sexual desire. While cases of PGAD/GPD in association with interstitial cystitis, urethritis, urinary tract infection, and urethral diverticulum have been described, bladder cancer has not yet been reported. To our knowledge, the present report describes the first known case of PGAD/GPD as the initial presentation of low‐grade papillary urothelial carcinoma of the bladder in a young female.

**Case Presentation:**

A 26 year‐old healthy female presented with a 3‐month history of constant, distressing clitoral engorgement, throbbing, and pain in her vulvar vestibule that began during masturbation. She also noted dysuria, urinary frequency, urgency, intermittent hematuria, and nocturia two to three times per night, pelvic floor hypertonicity, and restless legs. She denied any smoking history but did report a history of occasional resin exposure while making jewelry as a hobby. She underwent a bladder ultrasound that revealed a 7 × 5 × 6‐mm nonmobile lesion in the left posterior bladder wall, lateral to the ureteral orifice. Cystoscopy and pathology revealed low‐grade papillary (Ta) urothelial carcinoma of the bladder, and transurethral resection of bladder tumor was performed. Symptoms had completely resolved 3 months after the procedure, with continued use of topical lidocaine gel applied to the clitoris and pelvic floor physical therapy.

**Discussion:**

Our postulate to account for the constellation of symptoms tumor‐induced chronic irritation of the pelvic nerve resulting in reflexive bladder hypermotility, clitoral engorgement, and bowel dysfunction. In addition, central cross‐sensitization at the level of the sacral spinal cord could activate the postsynaptic neurons that normally respond to pudendal nerve afferent activity, resulting in clitoral and vestibular dysesthesia, reflexive pelvic floor hypertonicity via postsynaptic pudendal nerve efferents, and reflexive restless legs via postsynaptic sciatic nerve efferents. Further investigation is warranted to better understand the relationship between bladder tumors, PGAD/GPD, and related viscero‐visceral and viscero‐somatic reflex activity.

## 1. Introduction

Persistent genital arousal disorder/genito‐pelvic dysesthesia (PGAD/GPD) is a distressing condition characterized by unwanted genital arousal without associated sexual desire [1]. The presenting etiology and pathophysiology of PGAD/GPD are diverse. In 2021, the International Society for the Study of Women′s Sexual Health issued a consensus statement that characterized the condition based on the source of the symptoms across five regions: (1) end organ, (2) pelvis/perineum, (3) cauda equina, (4) spinal cord, and (5) brain. This framework has led to diagnostic and treatment approaches that are now considered the standard of care. Consequently, identifying the factors that contribute to PGAD/GPD allows for targeted treatment of the affected region(s).

Region 1, end organ dysfunction, can involve the clitoris, vestibule, vulva, vagina, and/or urinary tract [1]. Pelvic or perineal sources of PGAD/GPD symptoms encompass pelvic floor dysfunction, pelvic congestion syndrome, and pudendal neuropathy [1]. Symptoms can also arise from the cauda equina or spinal cord [1, 2]. Furthermore, neurological conditions including epilepsy, psychological factors including bipolar disorder, and changes in psychotropic medications have all been linked to PGAD/GPD [1]. Although region‐specific treatment algorithms exist, patients may have symptoms stemming from multiple regions, leading to extensive and often ineffective medical management. Central cross‐sensitization, a process in which pain in one body region generates pain perceived as originating in other body regions, can contribute to this widespread symptom presentation. In many instances, the etiology of PGAD/GPD remains unidentified, resulting in continued distress and stigma. While cases of PGAD/GPD associated with interstitial cystitis, urethritis, urinary tract infection, and urethral diverticulum have been described in the literature [1, 3], to our knowledge, PGAD/GPD secondary to bladder cancer has not yet been reported.

Bladder cancer predominantly affects older adults, with approximately 600,000 new cases reported globally in 2022 [4]. The disease is particularly uncommon in young females in the absence of traditional risk factors such as smoking or occupational chemical exposure [5]. When bladder cancer does occur in younger patients, most present with low‐grade, nonmuscle invasive bladder cancer (NMIBC) and demonstrate better outcomes compared with older patients [6]. Clinical presentation in this population can be highly variable, including symptoms of dysuria, macroscopic hematuria, or even more severe systemic symptoms [6, 7].

Although bladder cancer in young females is rare, its diagnosis can be particularly challenging due to atypical presentations that may mimic more common urogenital conditions [8]. Females in low risk categories may also face significant diagnostic delays due to disparities in treatments and representation in scientific literature [8]. Here, we report the first case known to us of PGAD/GPD associated with low grade papillary urothelial carcinoma in a young female patient with multiple risk factors. This contributes to the literature on clinical presentation of bladder cancer in a female patient as well as the differential diagnosis for patients presenting with persistent genital arousal symptoms.

## 2. Methods

With the patient′s permission, this case was brought to the attention of the Sexual Medicine Research Team (an international group of medical students, healthcare professionals, researchers, and patient advocates studying sexual medicine) during a monthly meeting. Ethics approval from the University of Saskatchewan and informed consent was obtained (Appendix A) to complete a retrospective chart review and a structured one‐on‐one interview including validated measures to quantify PGAD/GPD‐related distress and catastrophizing.

## 3. Case Presentation

### 3.1. Patient Information

A 26‐year‐old female with a history of hypertonic pelvic floor disorder and vulvodynia managed with pelvic floor physical therapy (PFPT), recurrent dysuria, restless legs syndrome, and anxiety (treated with cognitive behavioral therapy and sertraline) presented with a 3‐month history of persistent, distressing clitoral engorgement and throbbing. Symptoms began during masturbation and were accompanied by urinary urgency, frequency, intermittent hematuria, nocturia (two to three times/night), dysuria, and new‐onset constipation. Although her symptoms worsened with consumption of coffee and orange juice, they persisted despite elimination of these beverages and alcohol from her diet. She denied flank pain, fevers, and sensation of incomplete emptying. She described the genital sensation as a persistent urge to void accompanied by pelvic tingling, pressure, and burning, worsened by tight clothing or sitting, and associated with tingling radiation to her lower back and legs (similar to her restless legs but orgasmic in nature). She additionally reported provoked vestibular tenderness between the clitoris and urethra (12 o′clock position). The symptoms disrupted sleep and contributed to anxiety and depression. Upon interviewing the patient, validated questionnaire scores were notable for severe PGAD/GPD symptoms (Persistent Genital Arousal Sensations Questionnaire 60/60) (Appendix B), pain catastrophizing (Pain Catastrophizing Scale 47/52) (Appendix C), and anxiety and depression (Beck Anxiety and Depression Indices 47/63) (Appendix D).

She denied smoking and allergies but endorsed social alcohol use and minimal resin exposure. Family history was noncontributory aside from a great‐grandfather with bladder cancer at age of 88.

Prior management included PFPT and psychotherapy with minimal improvement. A prior urologist diagnosed PGAD/GPD, discontinued sertraline, initiated vortioxetine and gabapentin, and performed urethral dilation—all without symptomatic relief.

### 3.2. Clinical Findings

At initial presentation, urinalysis was positive for trace blood. However, she had a total of 24 urinalyses over the 6 years she was followed, of which two urinalyses were positive for leukocytes and none were positive for nitrites or microhematuria (> 3 RBCs/hpf) [9]. The patient requested a lumbosacral MRI but it did not show any obvious pathology that could account for her symptoms. Thus, sacral radiculopathy was ruled out. Neurogenital testing was not completed. A colonoscopy was also performed as part of the patient′s workup, but it was unrevealing. The patient pursued a second opinion after declining a recommendation for clitoridectomy.

### 3.3. Diagnostic Assessment

**Figure 1:**
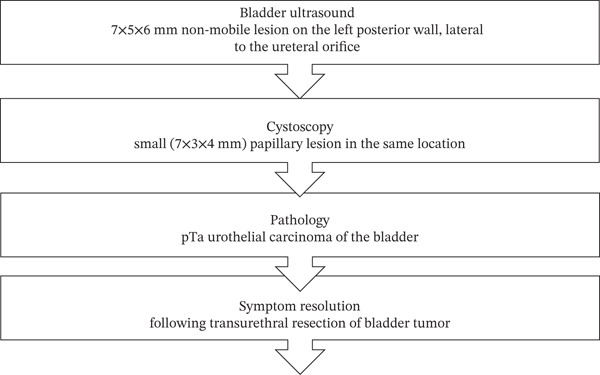
Timeline of diagnosis and therapeutic intervention.

**Figure 2:**
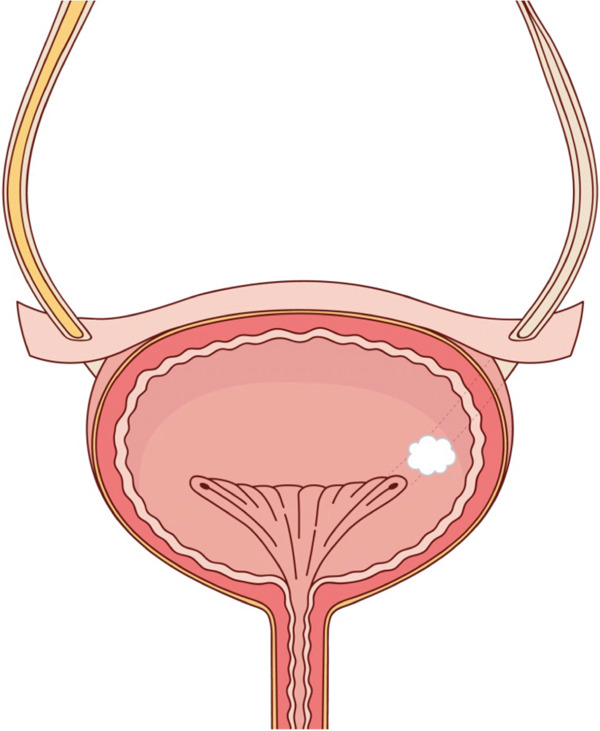
Cystoscopy visual. Cystoscopy revealed a small (7 × 3 × 4 mm) papillary lesion in the left posterior wall, lateral to the left ureteral orifice.

On further evaluation with bladder ultrasound, she was found to have a 7 × 5 × 6‐mm nonmobile lesion on the left posterior wall, lateral to the ureteral orifice (Figure 1). Further urological evaluation was recommended but bladder cancer was not on the radiologist′s differential, which included polyp, endometriosis, adenoma, inflammatory pseudotumor, and infectious cause. Renal ultrasound was unrevealing for hydronephrosis, stones, masses, or perinephric collections. Cystoscopy demonstrated a small (7 × 3 × 4 mm) papillary lesion in the same location (Figure 2).

### 3.4. Timeline

#### 3.4.1. Therapeutic Intervention

The papillary lesion was resected via transurethral resection of bladder tumor (TURBT). Pathology showed low‐grade, noninvasive papillary urothelial carcinoma (Ta). The patient resumed PFPT and was prescribed with lidocaine gel for external application to the clitoris for persistent clitoral throbbing and unwanted arousal.

### 3.5. Follow‐Up and Outcomes

Her PGAD/GPD symptoms fully resolved within 3 months. No recurrence was noted on surveillance cystoscopy at 3 and 9 months post‐TURBT [10]. Eight months following TURBT, she reported a return of clitoral throbbing, urinary urgency, and frequency. Cystoscopy showed no tumor recurrence but revealed an extensive squamous metaplasia in the left trigone. The area was fulgurated, given suspicion of symptom contribution. She subsequently experienced postvoid urethral throbbing lasting 15 min and a brief episode of hematuria. A negative urine culture was empirically treated with nitrofurantoin.

Two years following fulguration, she returned with recurrent dysuria and left‐sided lower back pain. Examination and laboratory evaluation, including urinalysis and urine culture, were unremarkable. She was treated with another empiric course of nitrofurantoin with partial subsequent symptom relief. Renal and pelvic ultrasound revealed normal kidneys and bladder, and two uterine fibroids (largest 1.4 cm). Repeat cystoscopy showed no recurrence. Her PGAD/GPD, pain catastrophizing, and depression scores had improved significantly, although she continued to endorse moderate depressive symptoms (PGASQ 18/60; PCS 19/52; BDI 28/63).

As of June 2025, her urinary, pelvic, and musculoskeletal symptoms have not returned and her bladder cancer has not recurred.

## 4. Discussion

### 4.1. Differential Diagnosis of PGAD/GPD

Interpretation of this case is limited by incomplete diagnostic evaluation and multiple overlapping interventions. Formal neurogenital and local anesthetic testing were not performed, limiting the ability to exclude peripheral causes such as pudendal neuropathy, clitorodynia, or pelvic floor dysfunction. This is notable given prior interventions, including PFPT and antidepressant changes, which may have influenced symptoms and raise the possibility that the bladder tumor was incidental. Nonetheless, although improvement in symptoms with topical lidocaine suggests a peripheral contribution, it does not rule out a downstream etiology.

Persistence of symptoms after sertraline discontinuation makes a Region 5 etiology less likely, though timing of washout relative to TURBT complicates interpretation. Lack of response to dietary modification and gabapentin argues against interstitial cystitis and primary neuropathic processes, and minimal prior benefit from PFPT suggests pelvic floor dysfunction alone is insufficient to explain her symptoms.

Despite incomplete exclusion of pudendal neuropathy (no diagnostic nerve studies or blocks were documented), sustained symptom resolution of the extensive constellation of seemingly unrelated symptoms following TURBT is consistent with a possible role of the bladder tumor as a visceral sensory trigger of central cross‐sensitization. This well‐characterized neurophysiological mechanism [11] could result in activation of postsynaptic neurons in the sacral spinal cord that characteristically respond to pudendal, pelvic, and sciatic nerve afferents and, reflexively, their motor components.

### 4.2. Cancer Risk Stratification

Initially, the patient′s providers attempted to rule out nonmalignant or gynecologic sources, including congenital abnormalities of the urinary tract. Given the absence of microhematuria on urinalysis, she was not considered to be at risk for bladder cancer [9, 10]. However, possible risk factors included gross hematuria without evidence of urinary tract infection on urinalysis, irritative voiding symptoms, and possible exposure to aromatic amines in dyes. Additional research on bladder cancer in the young female patient population may help to refine bladder cancer screening guidelines for rare presentations.

### 4.3. The Case for PGAD/GPD as a Manifestation of Bladder Cancer

**Figure 3:**
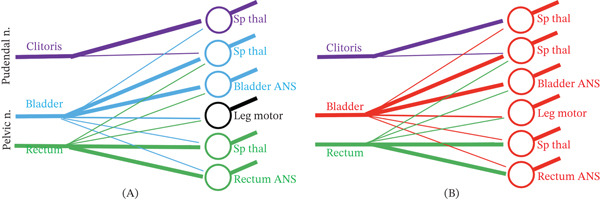
Postulated central cross‐sensitization of pelvic reflex pathways. (A) Under physiologic conditions, bladder and rectal afferents (thick lines) activate respective autonomic emptying reflexes and spinothalamic sensory pathways (Sp thal). Ancillary afferents (thin lines) between pelvic organs (bladder and rectum) and somatic pathways (legs and clitoris) typically are insufficient to activate shared postsynaptic neurons. (B) Pathological bladder irritation may amplify afferent activity as well as the ancillary fibers that share postsynaptic neurons. This may elicit abnormal sensations including clitoral dysesthesias and urinary urgency, as well as motor and autonomic responses including pelvic floor hypertonicity, restless legs, and constipation.

The patient′s reports of dysesthesia of the clitoris, urethra, bladder, lower back, and legs, and the motoric issues of restless legs, hypertonic pelvic floor, and urinary urgency and constipation, combined with a negative lumbosacral MRI, are consistent with central cross‐sensitization at the S2–S4 sacral spinal cord at the conus medullaris [12]. Chronic irritation arising from the bladder and urethra may intensify input via the pelvic nerve to the postsynaptic spinothalamic neurons in the sacral spinal cord. The underlying synaptic mechanisms include: (1) an increase in pelvic nerve pain neurotransmitters [13]; (2) sprouting of axon terminals in the pelvic nerve roots; (3) a decrease in inhibitory neurotransmitters in the involved synapses [14]; and (4) an increase in pain receptors in the postsynaptic neurons [13]. Consequent sensitization of these spinothalamic neurons could enable their activation by previously subthreshold adjacent pudendal and sciatic nerve afferents, thereby generating abnormal sensations in the clitoris, back, and legs (Figure 3). This is the phenomenon of central cross‐sensitization. [15]

Central cross‐sensitization may also involve spinal reflex circuits. Pudendal nerve input may generate pelvic floor hypertonicity, pelvic nerve input may generate urinary urgency and constipation, and the sciatic nerve input may generate restless legs. Resolution of these symptoms upon surgical removal of the bladder tumor may have contributed to the elimination of this proposed reflex pathway.

This case supports a potential association between PGAD/GPD and bladder cancer through central cross‐sensitization, wherein a Region 1 disease causes symptoms in Regions 1, 2, and 3. Noninvasive, papillary tumors may cause irritative bladder symptoms via pelvic afferents, with subsequent central cross‐sensitization in the conus medullaris to the pelvic, pudendal, and sciatic nerves.

## 5. Conclusion

This case highlights important implications for bladder cancer–risk stratification in young patients, particularly women presenting with atypical symptoms. Current screening paradigms may underrecognize malignancy risk in this population when classic markers such as microscopic hematuria are absent. This underscores the need for a more nuanced, symptom‐based approach to evaluation and suggests that early cystoscopic assessment may be warranted in select patients with persistent, unexplained lower urinary tract or pelvic sensory symptoms.

In addition, this case expands the clinical understanding of PGAD/GPD by illustrating a potential visceral etiology mediated through central cross‐sensitization. The convergence of pelvic, pudendal, and sciatic afferent inputs at the S2–S4 level provides a plausible mechanism by which a localized bladder lesion could generate broad symptomatology. Recognition of this mechanism has practical implications for diagnosis and management, as it encourages clinicians to consider nongenital sources of pathology in patients presenting with genital dysesthesia. Importantly, the complete resolution of symptoms following tumor resection is consistent with the potential reversibility of this sensitization process and highlights the importance of identifying and treating the primary visceral trigger. Given the speculative nature of this case, further study is warranted to determine the relationship between bladder cancer and PGAD/GPD.

## Funding

No funding was received for this manuscript.

## Consent

Patient gave informed consent for publication of de‐identified medical information via a modified BMJ Case Reports consent form [15]. The methodology received ethics approval from the University of Saskatchewan.

## Conflicts of Interest

The authors declare no conflicts of interest.

## Patient Perspective

The patient agrees with the report given of the case and expresses gratitude for bringing this to the attention of the medical community.

## Supporting information


**Supporting Information** Additional supporting information can be found online in the Supporting Information section. Appendix A: Consent form for case report adapted from BMJ Case Reports consent form [17]. B: Persistent Genital Arousal Sensations Questionnaire distributed to patient by researchers with responses [18]. C: Pain Catastrophizing Scale distributed to patient by researchers with responses [19]. D: Beck Depression Inventory distributed to patient by researchers with responses [20, 21].

## Data Availability

The data that support the findings of this study are available on request from the corresponding author. The data are not publicly available due to privacy or ethical restrictions.
