# Supplementary material for: Low‐Grade Papillary Urothelial Carcinoma of the Bladder Presenting as Persistent Genital Arousal Disorder/Genito‐Pelvic Dysesthesia: A Case Report
Source: Case Rep Urol. 2026 May 25;2026:3816720. doi: 10.1155/criu/3816720 (PMC13201904; doi:10.1155/criu/3816720)
Supplement: Supplementary file 1 — Supporting Information Additional supporting information can be found online in the Supporting Information section. Appendix A: Consent form for case report adapted from BMJ Case Reports consent form [17]. B: Persistent Genital Arousal Sensations Questionnaire distributed to patient by researchers with responses [18]. C: Pain Catastrophizing Scale distributed to patient by researchers with responses [19]. D: Beck Depression Inventory distributed to patient by researchers with responses [20, 21]. [file CRIU-2026-3816720-s001.docx]

**Appendix A**

**Consent form for case report adapted from BMJ Case Reports Consent Form^18^**

For a patient’s consent to publication of information about them in a journal or thesis

Name of person described in article or shown in photograph:__________________________

Subject matter of photograph or article: *PGAD/GPD masking bladder cancer*

Title of article: *Improvement of PGAD/GPD symptoms once bladder cancer was identified and treated*

Medical practitioner or corresponding author: *Dr. Shari McKinny, Dr. Rachel Rubin*

I_________________________________________ [insert full name] give my consent for this information about MYSELF OR MY CHILD OR WARD/MY RELATIVE [insert full name]:_________________________, relating to the subject matter above (“the Information”) to appear in a journal article, or to be used for the purpose of a thesis or presentation.

I understand the following:

1. The Information will be published without my name/child’s name/relatives name attached and every attempt will be made to ensure anonymity. I understand, however, that complete anonymity cannot be guaranteed. It is possible that somebody somewhere - perhaps, for example, somebody who looked after me/my child/relative, if I was in hospital, or a relative - may identify me.
2. The Information may be published in a journal which is read worldwide or an online journal. Journals are aimed mainly at health care professionals but may be seen by many non-doctors, including journalists.
3. The Information may be placed on a website.
4. I can withdraw my consent at any time before online publication, but once the Information has been committed to publication it will not be possible to withdraw the consent.

Signed:__________________________________ Date: ______________________

**OPTIONAL:**

Additionally, I give my permission to the researchers to record any interviews done for the purposes of transcribing the materials, after which any recordings will be deleted.

Signed:__________________________________ Date: ______________________

Signature of requesting medical practitioner/health care worker:

_____________________Date:______________

**Appendix B**

**Persistent Genital Arousal Sensations Questionnaire^19^**

1. I have experienced genital arousal (genital sensitivity, fullness or swelling) that is not related to any sexual excitement or desire (my genitals feel ‘turned on’ but my mind does not)

| 0 | 1 | 2 | **3** | 4 | 5 | 6 | 7 | 8 | 9 | **10** |
| --- | --- | --- | --- | --- | --- | --- | --- | --- | --- | --- |
| I do not experience  this |  |  |  |  | I experience this occasionally |  |  |  |  | I experience this often |

1. I have experienced genital arousal (genital sensitivity, fullness or swelling), which persists for an extended period of time (hours to days)

| 0 | **1** | 2 | 3 | 4 | 5 | 6 | 7 | 8 | 9 | **10** |
| --- | --- | --- | --- | --- | --- | --- | --- | --- | --- | --- |
| I do not experience  this |  |  |  |  | I experience this occasionally |  |  |  |  | I experience this often |

1. I have experienced genital arousal (genital sensitivity, fullness or swelling), which does not go away after an ordinary orgasm

| 0 | **1** | 2 | 3 | 4 | 5 | 6 | 7 | 8 | 9 | **10** |
| --- | --- | --- | --- | --- | --- | --- | --- | --- | --- | --- |
| I do not experience  this |  |  |  |  | I experience this occasionally |  |  |  |  | I experience this often |

1. I have experienced persistent (hours to days) genital arousal that I would describe as ‘unwanted’ or ‘intrusive’

| 0 | 1 | **2** | 3 | 4 | 5 | 6 | 7 | 8 | 9 | **10** |
| --- | --- | --- | --- | --- | --- | --- | --- | --- | --- | --- |
| I do not experience  this |  |  |  |  | I experience this occasionally |  |  |  |  | I experience this often |

1. I have experienced persistent (hours to days) genital arousal, and I do not know what the cause is

| 0 | 1 | 2 | 3 | 4 | 5 | 6 | 7 | 8 | 9 | **10** |
| --- | --- | --- | --- | --- | --- | --- | --- | --- | --- | --- |
| I do not experience  this |  |  |  |  | I experience this occasionally |  |  |  |  | I experience this often |

1. How would you describe your persistent genital arousal sensations? (If you selected ‘I do not experience this’ to all of the questions above, please select ‘Not Applicable’)

| 0 | 1 | 2 | 3 | 4 | 5 | 6 | 7 | 8 | 9 | **10 10** |
| --- | --- | --- | --- | --- | --- | --- | --- | --- | --- | --- |
| Enjoyable |  |  |  |  | Neutral |  |  |  |  | Distressing |

**Appendix C**

**Pain Catastrophizing Scale^20^**

**Pre in blue**

**Post in red**

**Pain/Catastrophizing**

**Pre: 47**

**Post: 19**

Everyone experiences painful/uncomfortable situations at some point in their lives. We are interested in the types of thoughts and feelings that you have when you are experiencing Persistent Genital Arousal Disorder/Genito-pelvic Dysaesthesia (PGAD/GPD) symptoms.

Listed below are thirteen statements describing different thoughts and feelings that may be associated with persistent genital arousal sensations. Using the following scale, please indicate the degree to which you have these thoughts and feelings when you are experiencing Persistent Genital Arousal Disorder/Genito-pelvic Dysaesthesia (PGAD/GPD) symptoms.

| Not at all | To a slight degree | To a moderate degree | To a great degree | All the time |
| --- | --- | --- | --- | --- |
| **0** | **1** | **2** | **3** | **4** |

______________________________________________________________

***When I’m experiencing Persistent Genital Arousal Disorder/Genito-pelvic Dysaesthesia (PGAD/GPD) symptoms …***

1. I worry all the time about whether the pain/sensations will end. **4 1**
2. I feel I can’t go on. **4 1**
3. It’s terrible and I think it’s never going to get any better. **4 1**
4. It’s awful and I feel that it overwhelms me. **4 2**
5. I feel I can’t stand it anymore. **4 1**
6. I become afraid that the sensations will get worse. **4 3**
7. I keep thinking of other painful events. **2 2**
8. I anxiously want the pain/sensations to go away. **4 2**
9. I can’t seem to keep it out of my mind. **4 1**
10. I keep thinking about how much it hurts. **4 1**
11. I keep thinking about how badly I want the sensations to stop. **4 2**
12. There’s nothing I can do to reduce the intensity of the pain/sensations. **3 0**
13. I wonder whether something serious may happen. **2 2**

PGASQ

**Pre: 60**

**Post: 18**

**Appendix D**

**Beck Depression Inventory^21,22^**

ANXIETY

Beck anxiety

**Pre: 16, low anxiety**

**Post: 7, low anxiety**

Numbness or tingling **3 2**

Feeling hot **1 0**

Wobbliness in legs **0 0**

Unable to relax **3 1**

Fear of worst happening **2 2**

Dizzy or lightheaded **0 0**

Heart pounding/racing **1 0**

Unsteady **0 0**

Terrified or afraid **2 0**

Nervous **3 1**

Feeling of choking **0 0**

Hands trembling **0 0**

Shaky / unsteady **0 0**

Fear of losing control **2 1**

Difficulty in breathing **0 0**

Fear of dying **0 0**

Scared **1 0**

Indigestion **0 0**

Faint / lightheaded **0 0**

Face flushed **0 0**

Hot/cold sweats **0 0**

DEPRESSION

Beck depression

**Pre= 47, indicates severe depression**

**Post= 28, indicates moderate depression**

1. Sadness **3 2**

0. I do not feel sad.

1. I feel sad much of the time.

2. I am sad all the time.

3. I am so sad or unhappy that I can't stand it.

2. Pessimism **2 2**

0. I am not discouraged about my future.

1. I feel more discouraged about my future than I used to.

2. I do not expect things to work out for me.

3. I feel my future is hopeless and will only get worse.

3. Past Failure **1 2**

0. I do not feel like a failure.

1. I have failed more than I should have.

2. As I look back, I see a lot of failures.

3. I feel I am a total failure as a person.

4. Loss of Pleasure **3 1**

0. I get as much pleasure as I ever did from the things I

enjoy.

1. I don't enjoy things as much as I used to.

2. I get very little pleasure from the things I used to enjoy.

3. I can't get any pleasure from the things I used to enjoy.

5. Guilty Feelings **1 2**

0. I don't feel particularly guilty.

1. I feel guilty over many things I have done or should

have done.

2. I feel quite guilty most of the time.

3. I feel guilty all of the time.

6. Punishment Feelings **3 1**

0. I don't feel I am being punished.

1. I feel I may be punished.

2. I expect to be punished.

3. I feel I am being punished.

7. Self-Dislike **3 3**

0. I feel the same about myself as ever.

1. I have lost confidence in myself.

2. I am disappointed in myself.

3. I dislike myself.

8. Self-Criticalness **2 2**

0. I don't criticize or blame myself more than usual.

1. I am more critical of myself than I used to be.

2. I criticize myself for all of my faults.

3. I blame myself for everything bad that happens.

9. Suicidal Thoughts or Wishes **1 1**

0. I don't have any thoughts of killing myself.

1. I have thoughts of killing myself, but I would not

carry them out.

2. I would like to kill myself.

3. I would kill myself if I had the chance.

10. Crying **2 1**

0. I don't cry anymore than I used to.

1. I cry more than I used to.

2. I cry over every little thing.

3. I feel like crying, but I can't.

11. Agitation **3 0**

0. I am no more restless or wound up than usual.

1. I feel more restless or wound up than usual.

2. I am so restless or agitated, it's hard to stay still.

3. I am so restless or agitated that I have to keep

moving or doing something.

12. Loss of Interest **3 1**

0. I have not lost interest in other people or

activities.

1. I am less interested in other people or things

than before.

2. I have lost most of my interest in other people or

things.

3. It's hard to get interested in anything.

13. Indecisiveness **2 1**

0. I make decisions about as well as ever.

1. I find it more difficult to make decisions than

usual.

2. I have much greater difficulty in making

decisions than I used to.

3. I have trouble making any decisions.

14. Worthlessness **3 2**

0. I do not feel I am worthless.

1. I don't consider myself as worthwhile and useful

as I used to.

2. I feel more worthless as compared to others.

3. I feel utterly worthless.

15. Loss of Energy **1 1**

0. I have as much energy as ever.

1. I have less energy than I used to have.

2. I don't have enough energy to do very much.

3. I don't have enough energy to do anything.

16. Changes in Sleeping Pattern **2b 2a**

0. I have not experienced any change in my sleeping.

1a I sleep somewhat more than usual.

1b I sleep somewhat less than usual.

2a I sleep a lot more than usual.

2b I sleep a lot less than usual.

3a I sleep most of the day.

3b I wake up 1-2 hours early and can't get back to

sleep.

17. Irritability **3 1**

0. I am not more irritable than usual.

1. I am more irritable than usual.

2. I am much more irritable than usual.

3. I am irritable all the time.

18. Changes in Appetite **2a 0**

0. I have not experienced any change in my

appetite.

1a My appetite is somewhat less than usual.

1b My appetite is somewhat greater than usual.

2a My appetite is much less than before.

2b My appetite is much greater than usual.

3a I have no appetite at all.

3b I crave food all the time.

19. Concentration Difficulty **2 1**

0. I can concentrate as well as ever.

1. I can't concentrate as well as usual.

2. It's hard to keep my mind on anything for

very long.

3. I find I can't concentrate on anything.

20. Tiredness or Fatigue **2 1**

0. I am no more tired or fatigued than usual.

1. I get more tired or fatigued more easily than usual.

2. I am too tired or fatigued to do a lot of the things I

used to do.

3. I am too tired or fatigued to do most of the

things I used to do.

21. Loss of Interest in Sex **3 1**

0. I have not noticed any recent change in my

interest in sex.

1. I am less interested in sex than I used to be.

2. I am much less interested in sex now.

3. I have lost interest in sex completely.
